# Full-length transcriptome sequences of ephemeral plant *Arabidopsis pumila* provides insight into gene expression dynamics during continuous salt stress

**DOI:** 10.1186/s12864-018-5106-y

**Published:** 2018-09-27

**Authors:** Lifei Yang, Yuhuan Jin, Wei Huang, Qi Sun, Fang Liu, Xianzhong Huang

**Affiliations:** Special Plant Genomics Laboratory, College of Life Sciences, Shihezi University, Shihezi, 832003 China

**Keywords:** *Arabidopsis pumila*, Salt tolerance, Differentially expressed genes, MAPK, SMRT

## Abstract

**Background:**

*Arabidopsis pumila* is native to the desert region of northwest China and it is extraordinarily well adapted to the local semi-desert saline soil, thus providing a candidate plant system for environmental adaptation and salt-tolerance gene mining. However, understanding of the salt-adaptation mechanism of this species is limited because of genomic sequences scarcity. In the present study, the transcriptome profiles of *A. pumila* leaf tissues treated with 250 mM NaCl for 0, 0.5, 3, 6, 12, 24 and 48 h were analyzed using a combination of second-generation sequencing (SGS) and third-generation single-molecule real-time (SMRT) sequencing.

**Results:**

Correction of SMRT long reads by SGS short reads resulted in 59,328 transcripts. We found 8075 differentially expressed genes (DEGs) between salt-stressed tissues and controls, of which 483 were transcription factors and 1157 were transport proteins. Most DEGs were activated within 6 h of salt stress and their expression stabilized after 48 h; the number of DEGs was greatest within 12 h of salt stress. Gene annotation and functional analyses revealed that expression of genes associated with the osmotic and ionic phases rapidly and coordinately changed during the continuous salt stress in this species, and salt stress-related categories were highly enriched among these DEGs, including oxidation–reduction, transmembrane transport, transcription factor activity and ion channel activity. Orphan, MYB, HB, bHLH, C3H, PHD, bZIP, ARF and NAC TFs were most enriched in DEGs; ABCB1, CLC-A, CPK30, KEA2, KUP9, NHX1, SOS1, VHA-A and VP1 TPs were extensively up-regulated in salt-stressed samples, suggesting that they play important roles in slat tolerance. Importantly, further experimental studies identified a mitogen-activated protein kinase (MAPK) gene *MAPKKK18* as continuously up-regulated throughout salt stress, suggesting its crucial role in salt tolerance. The expression patterns of the salt-responsive 24 genes resulted from quantitative real-time PCR were basically consistent with their transcript abundance changes identified by RNA-Seq.

**Conclusion:**

The full-length transcripts generated in this study provide a more accurate depiction of gene transcription of *A. pumila*. We identified potential genes involved in salt tolerance of *A. pumila*. These data present a genetic resource and facilitate better understanding of salt-adaptation mechanism for ephemeral plants.

**Electronic supplementary material:**

The online version of this article (10.1186/s12864-018-5106-y) contains supplementary material, which is available to authorized users.

## Background

Salinization and secondary salinization of land has taken place along with global environment deterioration, which constrain plant growth, secondary metabolism and crop production, and endanger food security [1, 2]. Although significant progresses have been made in deciphering the molecular mechanism underlying salt tolerance in plants, it is always a challenging task to cultivate salt-tolerant crop varieties [3–5].

Plants are sessile organism and have to cope with adverse environments, such as salt and drought stress. Under high salinity condition, plant absorbs large amount of sodium (Na^+^) and chloride (Cl^−^) from soil through root system, causing osmotic stress and ion toxicity as a result [1, 6–9]. To cope with adverse environments, plants have evolved various mechanisms to survive high-salt soils including ion homeostasis and compartmentalization [8–11].

The Xinjiang Uygur Autonomous Region (also called Xinjiang for short), located in the border area of northwest China, is mostly covered with uninhabitable desert. Ephemeral plant, a particular component of desert flora, can take advantage of rainwater and snowmelt in spring to rapidly germinate and complete their life-cycle in about two months [12]. In China, ephemeral plants are only distributed in north Xinjiang, and mainly grow in the southern margin of the Gurbantunggut Desert based on the Flora Xinjiangensis [13]. The desert scientists believe that they play important roles in windbreak and sand fixation, water and soil conservation, and microhabitat improvement, as a result attracting the concern of many desert scientists.

An ephemeral plant, *Arabidopsis pumila* has considerable adaptability to local semi-arid and semi-salinized habitats [3, 12, 13]. We previously constructed a normalized cDNA library and analyzed the potential roles of genes represented by stress-responsive expressed sequence tags [3, 14]. To date no genome size information has been reported in *A. pumila*, RNA-Seq is an ideal method to study gene expression profiles. However, no studies have yet reported a deep sequencing-based transcriptome profiling of *A. pumila* in response to salt stress.

Second-generation sequencing (SGS, or called next-generation sequencing) provides precise and comprehensive analysis of RNA transcripts for gene expression, and become an everyday tool to explore biological questions [15]. Research on transcriptome-wide responses under salt stress have been performed in several salt-tolerant plant species using SGS: *Populus pruinosa*, *Reaumuria trigyna*, *Suaeda fruticosa* and *Thellungiella salsuginea* [16–19]. Single-molecule real-time (SMRT) sequencing carried out in the Pacific Biosciences (PacBio, Menlo Park, CA, USA) sequencing platform provides a third-generation sequencing (TGS) technology, offering great improvement over SGS technologies on read lengths [20] and avoids the transcriptome assembly required for SGS [21]. SMRT sequencing technology has been used to characterize the complexity of transcriptomes in *Salvia miltiorrhiza* [22], *Zea mays* [23], *Fragaria vesca* [24], *Sorghum bicolor* [25] and *Phyllostachys edulis* [26]. In the present study, we combined SGS and SMRT sequencing to generate a full-length *A. pumila* transcriptome. We examined the profiles of differentially expressed genes (DEGs) of *A. pumila* leaves under salt stress, via a series of transcriptome SGS sequencings. In total, 59,328 transcripts were first obtained from *A. pumila*, and 8075 DEGs were detected during salt acclimation. These data provide a clear view of the transcriptomic dynamics for *A. pumila* in response to salt stress, and will facilitate future research towards elucidating the mechanisms of salinity adaptation of ephemeral plants.

## Results

### Physiological changes of *A. pumila* under salt stress

Since salt stress causes active oxygen damage to plants, we investigated the physiological change in *A. pumila* during the first 48 h of salt stress. There were different patterns of change for different physiological indexes (Additional file 1: Figure S1). No obvious difference was observed in chlorophyll content after 0.5 h of salt treatment, and this value began to increase at 3 h (Additional file 1: Figure S1A) and decreased at 12 h. Significantly higher contents of proline and malondialdehyde (MDA) were measured in salt-stressed plants. Proline content increased immediately after imposition of salt stress, and the rate of rise obviously accelerated after 24 h (Additional file 1: Figure S1B). Similarly, the MDA content also increased immediately after stress, but obviously accelerated after 3 h, peaked at 12 h and then declined (Additional file 1: Figure S1C). It should be noted that, under salt stress, the MDA content was still higher than control. The activity of superoxide dismutase (SOD), gradually increased after the onset of salt stress, increased significantly by 6 h reached a peak at 12 h and then declined (Additional file 1: Figure S1D), indicating that the reactive oxygen species (ROS) scavenging system should begin to play a role in response to salt stress. Leaf Na^+^ concentration increased gradually after salt stress and continued to rise (Additional file 1: Figure S1E), but there were only minor changes in potassium (K^+^) concentration during the 48 h of salt stress (Additional file 1: Figure S1F). These results indicate the complexity of *A. pumila* physiological changes in response to salt stress. Under high salt stress, cytosolic Na^+^ concentration increases rapidly in *A. pumila*, leading to cellular toxicity, an increase of MDA and inhibition of K^+^ absorption. At the same time, plant cell accumulate osmoprotectant such as proline or upregulate key enzyme for regulating reactive oxygen species such as SOD to balance the osmotic pressure of the ions to generate salt tolerance.

### Transcriptome sequencing

To identify and characterize the transcriptomes of *A. pumila* leaf tissues between control and salt-stress treatments, we employed joint the PacBio SMRT and SGS technologies for whole-transcriptome profiling. In total, Illumina sequencing yielded more than 1.15 billion clean reads (Additional file 2: Table S1). The analysis result is called ‘Illumina’ hereafter. SMART sequencing yielding 366,683 reads of inserts, of which 187,809 were full-length non-chimeric reads (containing 5′ primer, 3′ primer and the poly(A) tail) and 163,703 were non-full-length reads (Table 1). The average length of full-length non-chimeric read was 8978 bp.

**Table 1:** Statistics of SMRT sequencing data

Library	1–2 kb	2–3 kb	> 3 kb	All
No. of SMART cells	2	2	2	6
Number of reads of insert	137,729	116,608	112,346	366,683
Number of five prime reads	96,506	65,145	55,813	217,464
Number of three prime reads	99,750	67,970	57,879	225,599
Number of poly-A reads	99,430	66,614	57,281	223,325
Number of filtered short reads	7424	5992	1326	14,742
Number of non-full-length reads	44,531	55,812	63,360	163,703
Number of full-length reads	85,774	54,804	47,660	188,238
Number of full-length non-chimeric reads	85,502	54,681	47,626	187,809
Average full-length non-chimeric read length	1893	3017	4068	8978

To reduce the high error rates of the subreads, all 366,683 SMRT reads were corrected using the approximately 1.15 billion Illumina clean reads as input data (Additional file 2: Table S1). After error correction and removal of redundant transcript using the CD-HIT-EST program [22], a total of 59,328 non-redundant transcripts were produced, and each represented a unique full-length transcript of average length 2194 bp and N50 of 2717 bp. For simplicity, this result is called ‘SMRT’ hereafter.

Transcription length distribution of SGS and TGS results showed that approximately 52.8% of the assembled transcripts from Illumina reads were < 600 bases, whereas only 0.75% of the transcripts from the SMRT were < 600 bases (Additional file 3: Figure S2A). Of the assembled transcripts from SMRT, 4.2% were > 5000 bases but only 1.4% of the assembled transcripts from Illumina were > 5000 bases. Moreover, the average lengths of genes detected with SMRT were longer than for Illumina (Additional file 3: Figure S2B). Our results show that the SMRT sequencing provided a larger number of full-length and high-quality transcripts, and the use of SGS data to correct the low-quality SMRT reads improved PacBio long-read accuracy.

### Annotation and expression description of transcripts during salt stress

To acquire the most comprehensive annotation, all full-length transcripts from SMRT were aligned to public databases: NCBI non-redundant protein (NR) database, Swiss-Prot, Kyoto Encyclopedia of Genes and Genomes (KEGG), Protein family (pfam), EuKaryotic Ortholog Groups (KOG) and Gene Ontology (GO) by BLASTX; and NCBI nucleotide sequences (NT) by BLASTN (*E*-value <1e-5). A total of 58,664 (98.88%) genes from SMRT were annotated using the NR database. A Venn diagram showed that 19,988 genes were simultaneously annotated in NR, NT, pfam, GO and KOG databases (Additional file 4: Figure S3A). Based on homology with sequences of different species, 16,423 (30%) sequences were found against *Camelina sativa*, and 11,367 (20.7%) sequences had significant hits for *Capsella rubella*, followed by *A. lyrata* (11,281, 20.6%), *A. thaliana* (8531, 15.6%) and *Eutrema salsugineum* (1599, 2.9%). Only 10.2% of annotated sequences had similarity with other plant species (Additional file 4: Figure S3B).

To evaluate gene expression levels in response to salt stress, we mapped all Illumina clean reads of leaf tissues exposed to 0.5, 3, 6, 12, 24 and 48 h of salinity and controls assembled by Trinity [27] to the SMRT full-length transcriptome. Read-count for each gene was obtained from the mapping results by using RNA-Seq by Expectation Maximization software (Additional file 5: Table S2) [28]. The mappable read-count for each gene was then converted into expected number of fragments per kilobase of transcript sequence per million base pairs (FPKM) [29]. The total number of genes expressed at seven time points was 23,002 (38.8%) based on FPKM > 0.3, and these were selected for further analysis. The boxing diagram of FPKM indicated that gene expression levels were not evenly distributed for the different experimental conditions (Additional file 6: Figure S4A). For salt treatment, there was an obvious increase at 0.5 h compared to control, a decrease at 12 h, but a significant increase at 24 and 48 h. Among these genes, more than 10.6% had FPKM > 60 (Additional file 6: Figure S4B).

### Analysis of differentially expressed genes (DEGs)

**Fig. 1:**
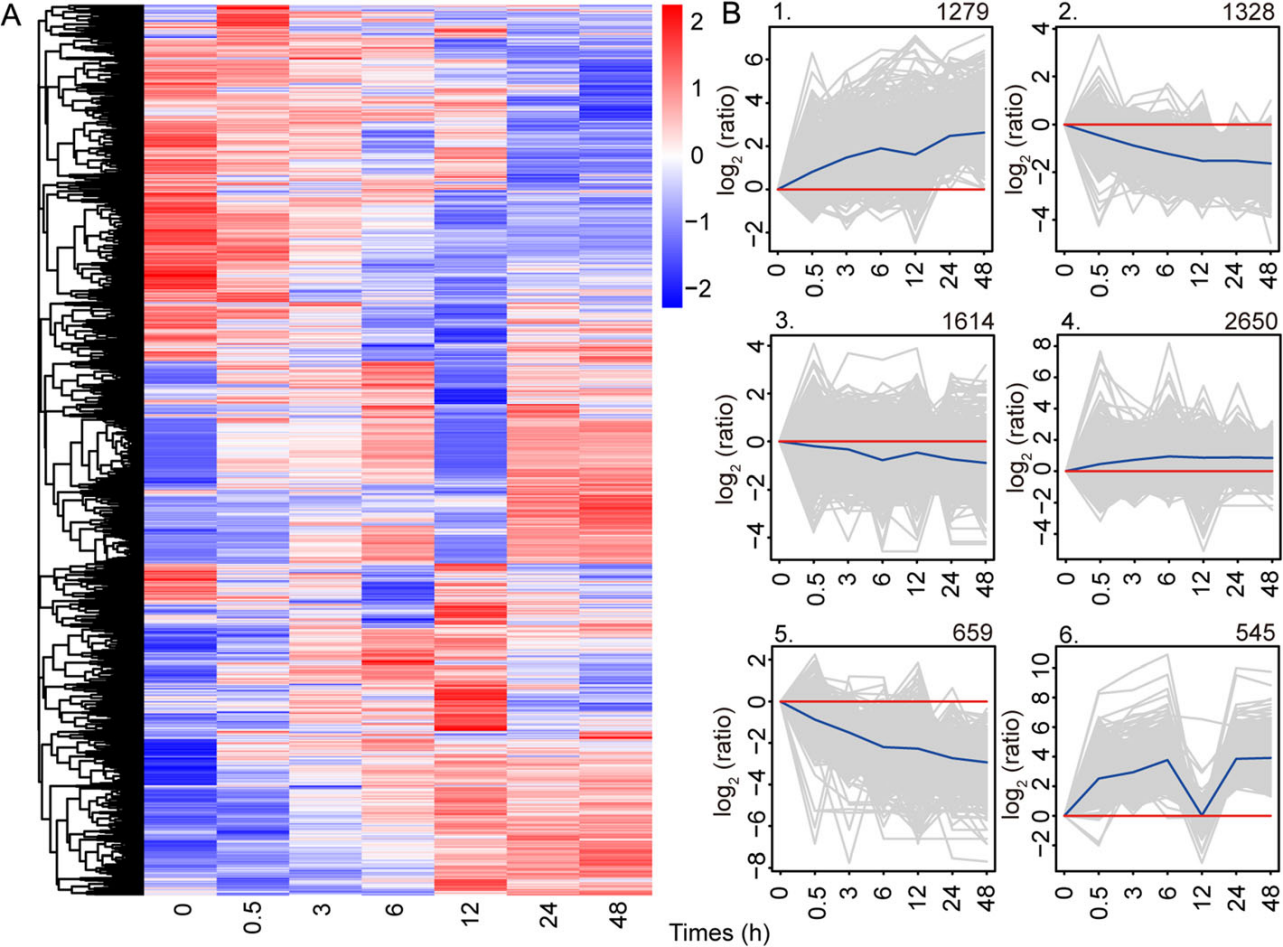
Clustering analysis of the differentially expressed genes (DEGs). **a**, Hierarchical clustering graph of the 8075 DEGs based on the averaged log_10_(FPKM+ 1) values of all genes in each cluster. **b**, The 8075 DEGs were clustered into six subclusters. Number of genes in each cluster is shown at the top of each cluster. Blue lines show the average values for relative expression levels in each subcluster; gray lines represent the relative expression levels of each gene in each cluster

In total, 8075 DEGs displaying up- or down-regulation between samples (adjusted *P* value of < 0.05) collected at any pair of time points were identified by comparing gene expression levels under salt treatment vs control conditions at 0 h (Additional file 7: Table S3). Clustering patterns of DEGs under different experimental treatments were determined by cluster analysis of all DEGs using the Euclidean distance method associated with complete linkage [18, 24] (Fig. 1a). This clustering pattern suggests that a set of genes was quickly activated during the early stage of salt stress (6 h), and other genes were activated with a longer period of stress; some of them were continuously and highly expressed during salt stress, but some returned to normal expression level. The 8075 DEGs identified were grouped into six subclusters with various temporal expression patterns (Fig. 1b). Genes in cluster 1 (1279 genes) and cluster 4 (2650 genes) were up-regulated at all times. Genes in cluster 1 were most strongly expressed during 0–6 h, gradually down-regulated during 6–12 h and then up-regulated. GO analysis of genes in cluster 1 revealed that most were associated with protein binding, transferase, ATP binding and catalytic activity. Genes in cluster 4 were gradually up-regulated during 0–6 h and then remained almost stable. These DEGs were similarly enriched in functional categories, such as protein binding and ATP binding, which indicates that these transcripts play important roles in salt tolerance in *A. pumila*. In contrast, genes in cluster 2 (1328 genes) and cluster 5 (659 genes) were down-regulated at all times. Transcripts in these two clusters function in similar pathway, such as oxidoreductase activity and hydrolase activity. Genes in cluster 3 (1614 genes) were also weakly down-regulated all times, gradually up-regulated during 6–12 h and then down-regulated. Genes involved in zinc ion binding and protein kinase activity were enriched in cluster 3. However, the expression patterns of genes in cluster 6 (545 genes) were more complicated: they were obviously up-regulated during 0–6 h, rapidly down-regulated at 6–12 h, and then strongly expressed during 12–24 h, and almost stable at 24–48 h. We noted that most genes function as catalytic activity and metal ion binding in cluster 6. This clustering pattern showed a complex dynamics of gene expression for DEGs, and allowed us to identify genes over multiple time points of *A. pumila* in response to salt stress.

### Comparison of DEGs between salt-stressed and control samples

**Fig. 2:**
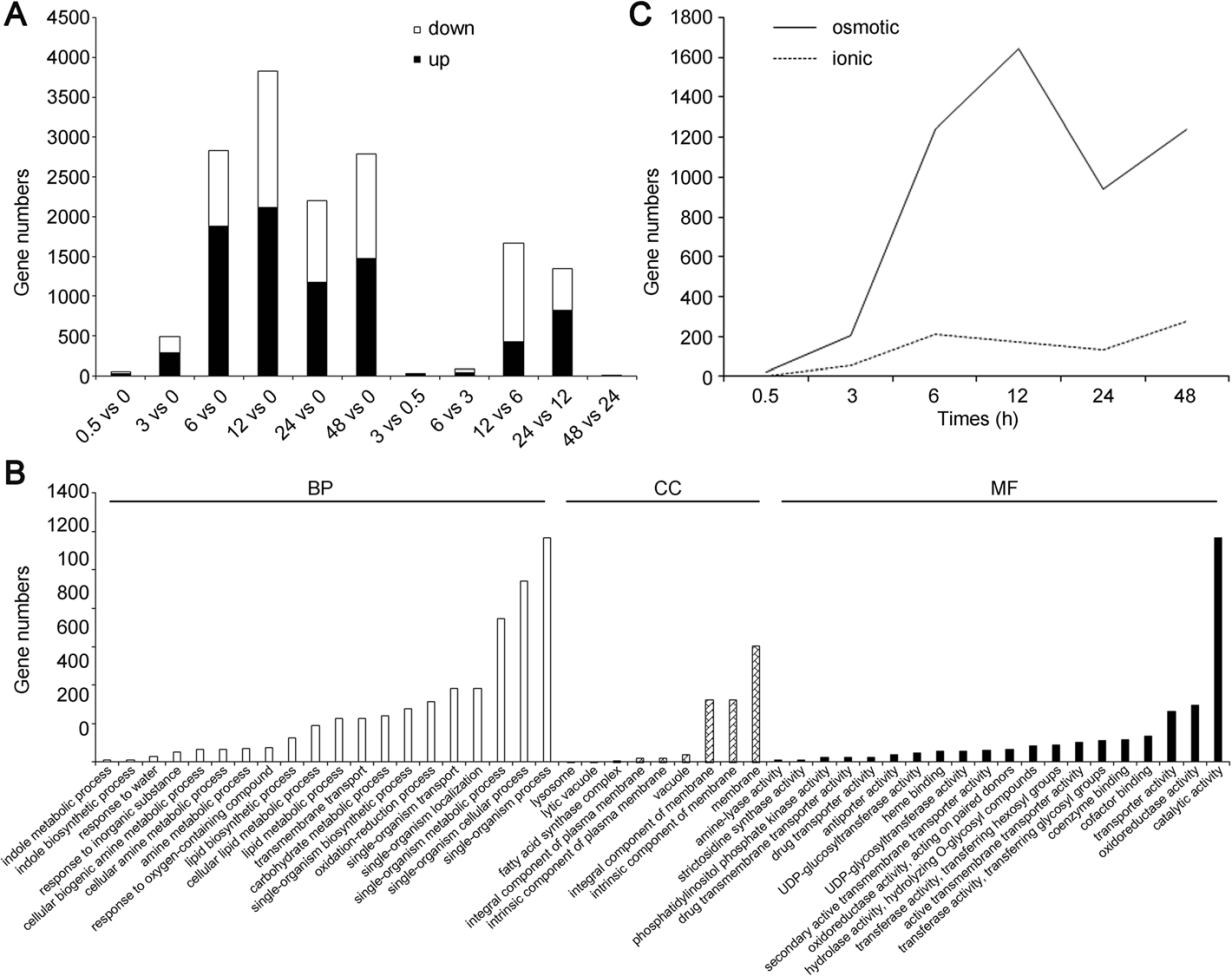
Summaries of differentially regulated genes during the time course. **a**, Differentially expressed genes were identified either by a comparison of each time point of salt stress to 0 h or comparing the adjacent stages. “0.5 vs 0” indicates a comparison between the gene expression in the 0.5-h stress with that in the 0-h stress, and this similarly applies to the other labels on the x-axis. **b**, Gene ontology classification of 2121 up-regulated DEGs at 12 h of salt stress. BP: biological process; CC: cellular component; MF: molecular function. **c**, Lines indicate changes in gene expression relating to the osmotic (solid line) and ionic phases (broken line) of salt stress, respectively. Genes involved in the osmotic phases are mainly associated with osmotic adjustment, osmolyte production, water loss and signal transduction pathways, etc. Genes identified in ionic phase are primarily responsible for ion transport and ion equilibrium [1, 7, 11, 30–32]

Compared with their expressions at 0 h, a number of DEGs showed a rising trend at the time points of 0.5, 3, 6, 12, 24 and 48 h in the whole salt treatment procedure (Fig. 2a). There were much fewer DEGs at 0.5 and 3 h of salt stress than at other times. Only 49 genes showing differential expression at 0.5 h of salt stress: 32 up-regulated and 17 down-regulated. There were 488 DEGs at 3 h of salt stress: 283 up-regulated and 205 down-regulated. However, the number of DEGs increased with the extension of treatment time. There were 2833 DEGs at 6 h of salt treatment, 3826 DEGs at 12 h, 2200 DEGs at 24 h and 2788 DEGs at 48 h. Of the 3826 DEGs at 12 h of salt stress, 2121 were up-regulated, and 1705 were down-regulated. 2121 up-regulated unigenes were assigned into the GO categories of biological process, cellular component and molecular function (Fig. 2b). “single-organism process”, “single-organism cellular process” and “single-organism metabolic process” were the most enriched terms of the biological process. In the cell component, “membrane”, “intrinsic component of membrane” and “integral component of membrane” were the most enriched, suggesting that cell membrane proteins and signaling molecules play crucial roles in response to salt stress in *A. pumila*. “catalytic activity” was the most enriched term of the molecular function, followed by “oxidoreductase activity” and “transporter activity”, suggesting multiple molecular pathway during the processes of salt stress in *A. pumila*. Overall, the functional and numerical changes in DEGs reflected the highly dynamic and coordinated changes in gene expression responses of *A. pumila* to a saline environment. Interestingly, more genes were up-regulated than down-regulated.

Osmotic stress and ion toxicity are considered to be the two major components of the plant salt-stress response [1, 6, 7]. Gene expression changes directly related to osmotic stress or ion function were summarized graphically (Fig. 2c) based on information on gene annotations (Additional file 7: Table S3) and on many published studies [1, 7, 11, 30–32]. There were 4425 DEGs associated with osmotic response during the whole processes of salt stress, only 680 DEGs associated with ionic response (Additional file 8: Table S4). The number of genes known to be related to the osmotic response was expressed rapidly at 3 h of salt stress, peaked at 12 h and subsided rapidly, and then ascend rapidly at 24 h. However, genes related to the ionic response gradually increased within 6 h of salt stress, then gradually declined and then rose at 24 H*. far* more genes were related to the osmotic phase than the ionic phase. However, gene expression relating to the two phases was almost simultaneous.

**Fig. 3:**
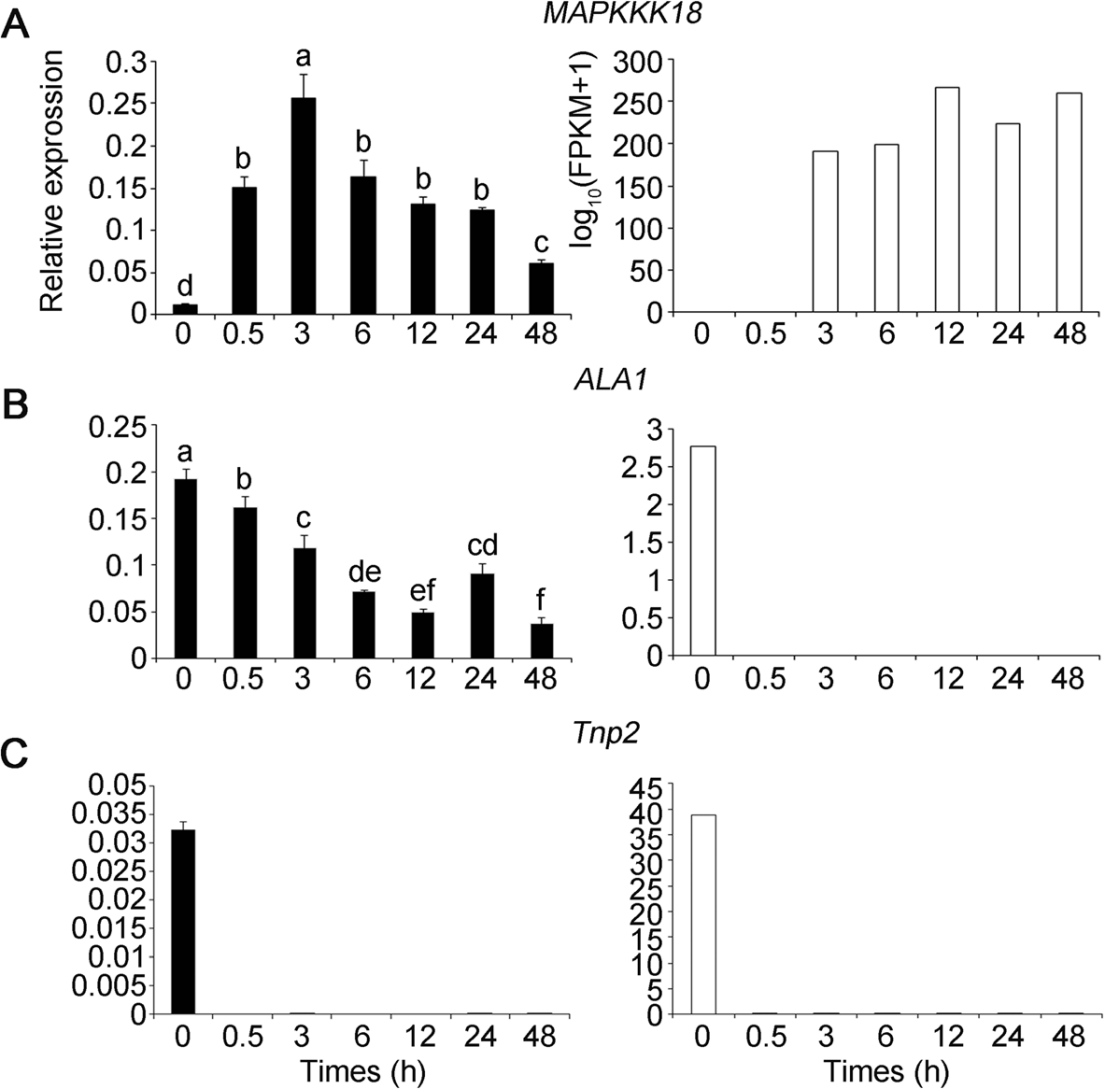
Expression pattern for the three differentially co-expressed genes throughout the entire period of salt treatment validated by qRT-PCR. **a**, *MAPPK18*. **b**, *ALA1*. **c**, *Tnp2*. The relative qRT-PCR expression level is shown on the left. The normalized expression (log_10_(FPKM+ 1)) of RNA-Seq is indicated on the right. Data are represented as mean ± standard error of the mean (S.E.M). Statistically significant differences are indicated by different lower-case letters (*P* < 0.05, Duncan’s multiple range tests)

We then analyzed the number of unique and shared DEGs between samples exposed to salt stress for 0.5, 3, 6, 12, 24 and 48 h, and control using Venn diagrams. Only three genes, *Mitogen-activated protein kinase kinase kinase 18* (*MAPKKK18*), *aminophospholipid ATPase 1* (*ALA1*) and CACTA-like transposase family (*Tnp2/En/Spm*) gene (Additional file 9: Table S5), were differentially co-expressed at all six time points of salt stress. Furthermore, quantitative real-time PCR (qRT-PCR) confirmed that *MAPKKK18* was up-regulated, whereas *ALA1* and *Tnp2* were down-regulated at all six time points, which was highly consistent with the SMRT sequencing results (Fig. 3).

**Fig. 4:**
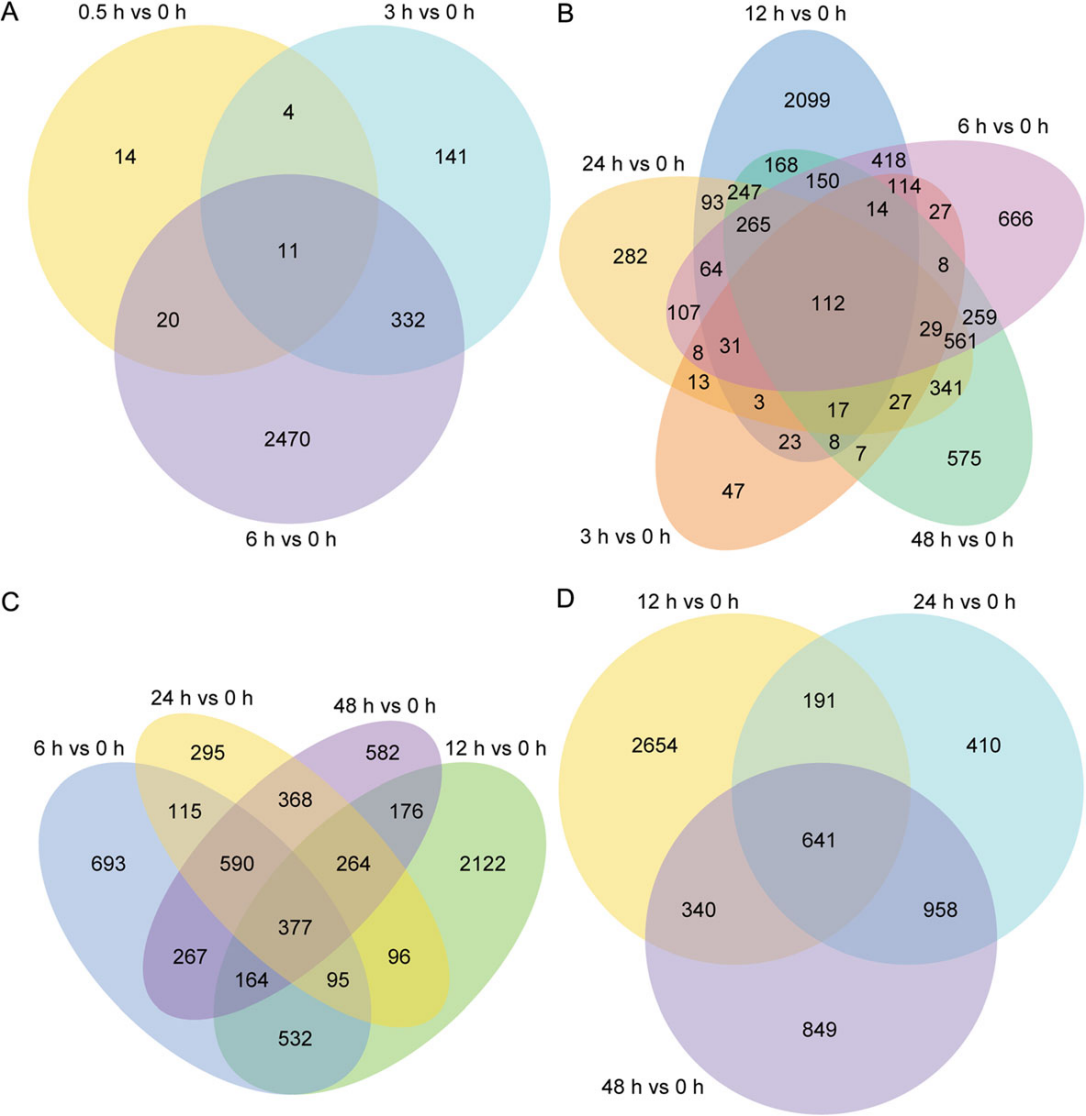
Venn diagrams describing the numbers of unique and shared differentially expressed genes (DEGs) between samples exposed to salinity stress for 0.5, 6, 12, 24 and 48 h, and control. **a**, DEGs at 0.5, 3 and 6 h. **b**, DEGs at 3, 6, 12, 24 and 48 h. **c**, DEGs at 6, 12, 24 and 48 h. **d**, DEGs at 12, 24 and 48 h

Only 11 genes were differentially co-expressed within 0–6 h of salt stress (Fig. 4a), but more genes were differentially co-expressed with prolonged salt stress. For example, 112 transcripts were differentially co-expressed within 3–48 h (Fig. 4b), 377 transcripts were differentially co-expressed within 6–48 h (Fig. 4c), and 641 genes were differentially co-expressed within 12–48 h (Fig. 4d). The GO term annotations revealed that the 377 DEGs were highly enriched in molecular functions categories: tetrapyrrole binding, heme binding, antioxidant activity, oxidoreductase activity, peroxidase activity and catalase activity (Additional file 10: Figure S5).

Furthermore, 377 DEGs were mapped onto 64 KEGG pathways to further validate the molecular functions and biological pathways. After multiple testing corrections, we chose the 20 most enriched pathways (based on adjusted *P*-value ≤0.05) to draw a scatter plot. Peroxisome, tryptophan metabolism, and glyoxylate and dicarboxylate metabolism were most enriched, followed by plant hormone signal transduction, starch and sucrose metabolism, photosynthesis-antenna proteins and phenylpropanoid biosynthesis (Additional file 11: Figure S6). These pathways have been reported to play roles in salt tolerance of plants [33, 34]. These characteristics and findings for the *A. pumila* transcriptome may facilitate the deciphering of the salinity adaptation machinery and allele mining of salt tolerant genes.

### Identification and expression patterns of putative transcription factors (TFs) and transporter proteins (TPs)

**Fig. 5:**
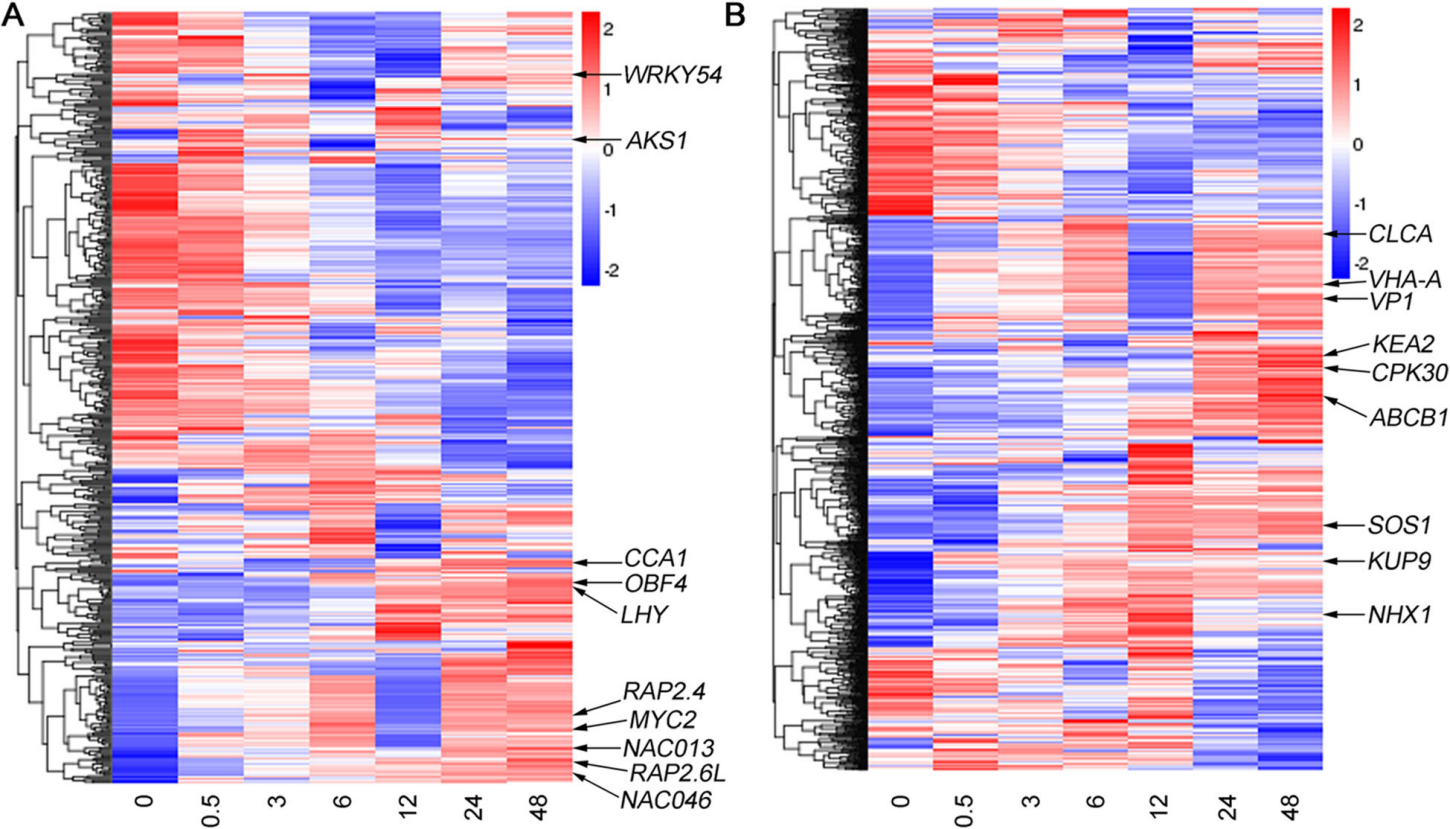
Expression patterns of transcription factors and transporter proteins. The heat map showing log_10_(FPKM+ 1) values of individual 483 transcription factors (**a**) and 1157 transporter proteins (**b**)

TF control the expression of numerous genes, and thus regulate many biological pathways including salt-related processes [35]. In the present study, 483 TF genes were differentially expressed between different time points, and were classified into 50 TF gene families according to PlantTFDB 2.0 (Additional file 12: Table S6 [35]). The most abundant TF family was the Orphans group (49 genes, 10.14%), followed by MYB (36, 7.45%), HB (31, 6.42%), bHLH (28, 5.8%), C3H (23, 4.76%), PHD (21, 4.35%), bZIP (20, 4.14%), ARF (19, 3.93%) and NAC (19, 3.93%). The heat map of gene expression illustrated that some of these TF genes were extensively up-regulated in response to salt stress in *A. pumila* (Fig. 5a), such as *MYB* (*CCA1* and *LHY*), *bZIP*s (*OBF4*), *AP2/ERF* (*RAP2.4* and *RAP2.6 L*), *NAC* (*NAC013* and *NAC046*), *bHLH* (*MYC2* and *AKS1*) and *WRKY54*. Dynamic changes of gene expression associated with these TFs may reveal their vital functions in plant salt tolerance. TPs function in moving other materials within an organism, such as carrier proteins and vesicular proteins [18, 36]. Among all DEGs, those for 1157 transporter proteins were selected for further analysis according to TransportDB (Additional file 13: Table S7) [36]. The heat map of TP genes expression showed that those encoding a vacuolar Na^+^/proton (H^+^) antiporter (NHX1), a plasma membrane-localized Na^+^/H^+^ antiporter (SOS1), a potassium ion transmembrane transporter (KUP9), a K^+^/H^+^ antiporter 2 (KEA2), a member of the voltage-dependent chloride channel (CLC-A), a Calcium-dependent protein kinase (CPK30), a vacuolar ATP synthase (VHA-A), a vacuole H^+^-inorganic pyrophosphatase (VP1) and an ATP-binding cassette (ABCB1) were also extensively up-regulated in salt-stressed samples (Fig. 5b). These results indicated that genes responsible for ion transport and energy balance are strongly activated in response to salt stress, and these maintain or re-establish homeostasis in the cytoplasm.

### Validation of SMRT expression patterns by qRT-PCR analysis

The candidate DEGs associated with salt-related processes were selected for qRT-PCR assays to validate the SMRT sequencing results. In addition to *MAPKKK18*, *ALA1* and *Tnp2*, we selected another 21 genes from the top 10 DEGs at one or more time points under salt-stress conditions. We noticed that the fold-changes in their expression calculated by sequencing did not exactly match with the expression values detected by qRT-PCR, but the expression profiles were basically consistent for all 21 genes (Additional file 14: Figure S7). These analyses confirmed the reliability of the gene expression values generated from SMRT sequencing results.

## Discussion

SGS technology has dramatically accelerated transcriptome research during recent decades, and short reads generated by SGS are highly accurate. However, short reads reduce the accuracy of sequence assembly and make bioinformatics analyses difficult [15, 37, 38]. The single-molecule long-read sequencing from PacBio greatly facilitates the de novo assembly of transcriptome in higher organisms [39, 40]. Although TGS has relatively high error rate, this shortcoming can be overcome through correction using short and high-accuracy SGS reads [37, 38]. Therefore, a hybrid sequencing approach combining the short and long-read sequencing technologies could provide high-quality and more complete assemblies in transcriptome studies, and this has been well documented [22, 39–41]. In the present study, we combined SGS and SMRT sequencing to generate a more complete *A. pumila* transcriptome. Our SMRT data were of high quality. The average full-length of reads of inserts was long enough to represent the full-length transcripts (Table 1). Furthermore, correction of the SMRT long reads using Illumina short reads led to high-quality full-length transcripts, reducing mis-assemblies of genes and gene families with high sequence identity.

### Gene expression dynamics of *A. pumila* in response to continuous salt stress

Several recent studies have reported transcript dynamics of different plant species in response to continuous salt stress [17, 18, 42–46], but few studies addressed more than six time points of salt stress. In our study, there were 8075 DEGs identified as responding to salt stress at all time points, but much fewer genes were differentially co-expressed for all time points. In fact, only three genes were differentially co-expressed at all six time points (Additional file 9: Table S5) and 641 were differentially co-expressed at three later time points (12, 24 and 48 h) (Fig. 4). The number of DEGs was most at 12 h of salt stress. The 2121 up-regulated DEGs were highly enriched in GO functional categories, including catalytic activity, oxidoreductase activity and transporter activity (Fig. 2b). This information will be useful to explore salt-tolerance mechanisms and mine new salt stress-related genes specific to *A. pumila*.

Comparison of DEGs between the salt-stressed and control samples indicated few DEGs within 30 min following imposition of salinity stress, but a number of genes were up- or down-regulated within 6 h (Fig. 2a). The largest numbers of DEGs occurred at 12 h during the 48 h of salt treatment. Comparison of DEGs between adjacent stages also indicated that a number of genes were differentially expressed at 12 or 24 h, and only eight genes were differentially expressed between 24 and 48 h time points of salt treatment. Most DEGs occurred during the first day of salt treatment, suggesting that we should pay more attention to the gene expression changes during the first 24 of salt stress in *A. pumila*. Identification of DEGs at six time points of salt treatment over two days provided comprehensive transcriptome dynamics of *A. pumila*, and also enhanced our understanding of molecular mechanisms of plant salt adaptation.

Long-term exposure to continuous salt-stress condition causes osmotic stress and ionic toxicity [1, 10]. A number of studies have analyzed gene expression changes in response to salt stress and reported expressed change in a large number of genes associated with osmotic and ionic response. Our study also identified relatively large numbers of genes associated with osmotic stress response in 2 days of salt stress, but relatively fewer genes were associated with ionic stress response (Fig. 2c). The DEGs were primarily responsible for osmotic adjustment and ionic response, including water balance, cell turgor maintenance, accumulation of soluble sugars, oxidoreductase activity, carbohydrate metabolic process, transmembrane transport and cation:sugar symporter (Additional file 8: Table S4). Furthermore, genes showing altered expression associated with the osmotic and the ionic phases simultaneously occurred within 0.5 h of exposure of plants to salinity, which were different previous reports [11]. Our study revealed that under salt stress conditions, more osmosis-related genes were up-regulated in *A. pumila*, endowing it with more salt tolerance.

### Identification of genes responsible for salt response

Plant adaptation or tolerance to salt stress involves complex molecular or genetic networks. Global analysis of stress-responsive genes facilitates understanding of the plant response to salt stress. In this study, identification and molecular function analyses of the 8075 DEGs reflected general gene expression changes in response to continuous salinity stress in *A. pumila* and provided a basis for further studies.

Several enriched biological processes, metabolic pathways and biochemical activities were identified based on GO and KEGG enrichment analysis of DEGs, providing gene expression overview underlying the salt-stress response in *A. pumila*. For example, many DEGs were enriched in GO terms such as single-organism metabolic process, oxidation–reduction process, carbohydrate metabolic process, transmembrane transport biological process, catalytic activity, oxidoreductase activity and transporter activity (Fig. 2b and Additional file 10), suggesting that maintaining membrane integrity and osmotic balance play vital roles in salt-stress tolerance in *A. pumila*. This information will be useful in elucidating salt-tolerance mechanisms and for mining new salt-stress related genes specific to *A. pumila*. Proline plays a crucial role in oxidative and osmotic responses in higher plants, and proline accumulation is a well-known measure adopted for alleviation of salt stress. We found that genes involved in arginine and proline metabolism were induced under stress, such as *P5CS1* (Additional file 11: Figure S6), which was consistent with our previous report [14]. Arginine and proline metabolism is one of the central pathways for the biosynthesis of the amino acids arginine and proline. Arginine is a kind of free amino acids, and decreases when exposed to salt stress, whereas proline concentration rises in response to salt stress [1, 9, 30]. Under salt stress conditions, osmoprotectants, such as proline and glycine betaine, must accumulate to balance the osmotic pressure of intracellular ions and provide tolerance towards stress. Measurement of physiological responses showed that proline content increased significantly (from 789 to 10,150 μg g FW^− 1^) with prolonged salt stress, which confirmed reliability of our transcriptome analysis results.

In plants, MAPK signaling networks has important roles in numerous biological processes, including cell division, development, hormone response, ROS homeostasis, senescence, as well as biotic and abiotic stress responses [47–53]. Expression of *Arabidopsis MAPKKK20* was up-regulated with NaCl treatment, and transgenic plants overexpressing *MAPKKK20* displayed tolerance to salt stress [54]. *Arabidopsis MAPKKK18* was shown to negatively regulate stomatal opening and positively regulate drought stress resistance [51, 52, 55]. Our results showed that *MAPKKK18* was rapidly up-regulated within 0.5 h of salt stress and was also continuously and highly expressed during 2 days of salt stress (Fig. 3). Although significant progress has been made in exploring how *MAPKKK18* responds to drought stress and abscisic acid, the detailed biological functions correlated with salt tolerance remain unclear. Its continuous high expression indicated that *MAPKKK18* may play vital roles in salt tolerance in *A. pumila*.

### Genes encoding TFs and TPs in response to salt stress

In plants, many transcription factors have been identified to confer salt tolerance using transcriptomic approaches, including AP2-EREBP, bHLH, bZIP, C2H2, NAC and WRKY [14, 56–58]. In this study, 480 TFs were differentially regulated under salt stress, suggesting that TFs play important roles in modulating the acclimation response of *A. pumila* to salt stress. Among these TFs, Orphans was most abundant, followed by MYB and HB TF families. The top 20 enriched TFs also include bHLH, bZIP, NAC, C2H2, WRKY and AP2-EREBP TF families (Additional file 12: Table S6). Gene expression analysis confirmed that many of them were up-regulated during the salt-stress treatment (Fig. 5a).

In plants, transporter proteins play crucial roles in fundamental processes such as uptake of nutrients, the efflux of toxic and other compounds and in ion homeostasis [43, 59]. In the current study, we predicted that 1157 genes for TPs or regulating ion homeostasis were differentially expressed in response to salt stress (Additional file 13: Table S7). The ATP-binding cassette transporters (ABC transporters) are members of a transport system superfamily whose main function is to mediate the energy-driven transport of many substrates, ranging from ions to macromolecules, across membranes [60, 61]. We found that the ABC G-type subfamily was the most abundant type identified in *A. pumila* in response to salt stress. Furthermore, expressions of genes encoding ion transporters or involved in homeostasis, such as *ABCB1*, *CLC-A*, *CPK30*, *KEA2*, *KUP9*, *NHX1*, *SOS1*, *VHA-A* and *VP1*, were up-regulated in salt-stressed samples (Fig. 5b). These results revealed that, in *A. pumila*, genes responsible for ion transport and homeostasis were strongly activated in responses to salt stress, hinting that they maybe corporate to orchestrate ion homeostasis in the cytoplasm to cope with osmotic imbalance and ion toxicity.

The differential expression patterns of the TFs and TPs in our study are consistent with transcriptomic profiles of other plant species under salt stress [18, 43, 44, 46, 62]. However, the present study was the first to comprehensively study the transcriptomic responses using six time points of continuous salt stress by integrating short-read sequencing and long-read SMRT sequencing technologies. The dynamic changes in DEGs of *A. pumila* provide insights into the mechanism underlying ephemeral plant adaptation to a saline environment. The genes identified in the present study may be suitable targets for biotechnological manipulation to improve plant salt tolerance.

## Conclusions

We explored transcriptomic changes in the ephemeral plant *A. pumila* in response to continuous salt stress, using integration of SGS and TGS technologies. Fifty-nine thousand three hundred twenty-eight unique full-length transcripts were generated, and 8075 DEGs were identified in the present study that are involved in carbohydrate metabolism, ion transporters, osmotic regulation and oxidation–reduction process in response to salt stress of this species. Most DEGs were activated within 6 h of salt stress, and the number of DEGs was greatest at 12 h. Genes associated with osmotic adjustment, ionic equilibrium, TFs and TPs were characterized in this study. In addition, only *MAPKKK18* gene was found to be continuously up-regulated during the whole process of salt treatment, suggesting its crucial role in salt tolerance in *A. pumila*. The availability of full-length transcripts generated in this study provides a more accurate depiction of gene transcription, and facilitates understanding of the salt-adaptation mechanism for *A. pumila*.

## Methods

### Plant materials, cultivation and salinity treatment

Seeds of *A. pumila* were collected in May 2012 from the southern margin (44°20’N and 87°46′E) of the Gurbantunggut Desert in Xinjiang, China. Collection of plant specimens every year is permitted by the Xinjiang Uygur Autonomous Region government, and sampling collection at these locations did not involve protected or endangered species. Seeds of *A. pumila* were surface sterilized and planted as described by Huang et al. (2017) [14]. After 7 days, the seedlings were transplanted into pots containing peat soil and vermiculite (1:1) and kept in a plant growth chamber with long day conditions (16-h-light/8-h-dark photoperiod, light intensity of 200 μmol photons m^− 2^ s^− 1^, ambient temperature of 22 °C). The six-week-old seedlings were transferred to a 100-mL conical flask containing Hoagland’s nutrient solution (1 mM MgSO_4_, 1 mM KH_2_PO_4_, 1 mM NH_4_NO_3_, 0.5 mM CaCl_2_, 0.1 mM FeNa-EDTA, 25 mM NaCl, 0.1 mM H_3_BO_3_, 0.1 mM Na_2_SiO_3_, 1.5 μM CuSO_4_, 50 μM KCl, 10 μM MnSO_4_, 0.075 μM Na_2_MoO_4_ and 2 μM ZnSO_4_) for 24 h, and then transferred to fresh Hoagland’s nutrient solution supplemented by 50 mM NaCl. The NaCl concentration in Hoagland’s nutrient solution was gradually increased by 50 mM NaCl per day until reaching 250 mM. Tissues of young leaves of salt-stressed plants were separately harvested at time points of 0.5, 3, 6, 12, 24 and 48 h of salt stress. Leaf samples from untreated plant were collected for RNA extraction as controls. We collected samples of seven time points in this study. The assays were conducted in three biological replicates, and each replicate represented one single plant. Altogether, 21 plants were sampled and immediately frozen in liquid nitrogen after collection and stored at − 80 °C for follow-up experiments.

### Measurement of physiological variables

Chlorophyll content was measured as described by Li et al. (2015) [63]. MDA content was determined according to Li et al. (2013) [64]. Free proline content and ion contents were determined according to the method of Wu et al. (2017) [62]. An atomic absorption spectrophotometer (Shanghai Precision & Scientific Instrument Co., Shanghai, China) was used in this study to quantify Na^+^ and K^+^ content.

### RNA isolation, quantification and qualification

Total RNA of leaves from *A. pumila* seedlings, sampled at different time points used for transcriptome sequencing, were extracted using RNAprep Pure Plant Kit (Tiangen Biotech, Beijing, China) and treated with RNase-free DNase (TIANGEN) following the manufacturer’s instructions. RNA degradation and contamination was monitored on 1% agarose gels. RNA purity was checked using a Nanodrop ND-1000 spectrophotometer (NanoDrop Technologies, Wilmington, DE, USA). The integrity of RNA samples was assessed using the RNA Nano 6000 Assay Kit of the Agilent Bioanalyzer 2100 system (Agilent Technologies, Palo Alto, CA, USA). RNA concentration was measured using Qubit® RNA Assay Kit in Qubit® 2.0 Flurometer (Life Technologies, Carlsbad, CA, USA).

### PacBio Iso-Seq library preparation and TGS

Equal amounts of RNA (1 μg per sample) from 21 single plants were pooled together, and then 3 μg RNA from it was used to prepare SMRT libraries. Three Iso-Seq libraries (1–2, 2–3 and 3–6 kb) were prepared according to the Isoform Sequencing protocol (Iso-Seq™) using the Clontech SMARTer PCR cDNA Synthesis Kit and the BluePippin Size Selection System (Sage Science, Beverly, MA) protocol as described by PacBio (P/N100–377–100-05 and P/N100–377–100-04). Briefly, after synthesis of first strand, the large-scale double-strand cDNA was generated with 12 PCR cycles using Phusion DNA polymerase (NEB, Beverly, MA, USA). The amplification program consists of 2 min of initial denaturation at 95 °C, followed by 12 cycles of 20 s at 98 °C, 15 s at 65 °C and 4 min at 72 °C, final 4 min of extension at 72 °C. Amplification was followed by size selection using the BluePippin (Sage Science, Beverly, MA, USA) of the following bins for each sample: 1–2, 2–3, and 2–6 kb. After size selection, another amplification was performed using 12 PCR cycles of the above amplification conditions. The amplified and size selected cDNA products were made into SMRTbell Template libraries according to the Iso-Seq protocol reference above.

A total of six SMRT cells were used for the three libraries at three size ranges: namely 1–2, 2–3, and > 3 kb. Libraries were subsequently sequenced using a Pacific Biosciences (PacBio) RS sequencing instrument with total six SMRT cells: the 1–2 and 2–3 kb libraries were sequenced using two SMRT cells, respectively; and the above 3 kb library using two SMRT cells.

### Illumina cDNA library preparation and SGS

A total of 1.5 μg of RNA per sample was used as input material for RNA sample preparations. Sequencing libraries were generated using a NEBNext® Ultra™ RNA Library Prep Kit for Illumina® (NEB) following the manufacturer’s recommendations, and index codes were added to attribute sequences to each sample. In brief, mRNA was purified from total RNA using poly-T oligo-attached magnetic beads, fragmented and used for cDNA synthesis with random hexamer primer (NEB). After end repair, adenylation, adapter ligation, cDNA purification, and PCR amplification, 21 paired-end cDNA libraries were constructed, and their qualities assessed on the Agilent Bioanalyzer 2100 system. After cluster generation, the library preparations were sequenced on an Illumina HiSeq 2500 platform (Illumina, San Diego, CA, USA) and paired-end reads were generated. High-throughput sequencing (both TGS and SGS) in this study was performed in the Novogene Bioinformatics Institute (Novogene, Beijing, China).

### Quality control and transcriptome assembly

Raw Illumina SGS reads of fastq format were first processed using in-house perl scripts. Clean reads were obtained by removing reads containing adaptors, reads containing poly-N and low-quality reads from raw data. At the same time, Q20, Q30, GC-content and sequence duplication level of the clean data were calculated. Transcriptome assembly was accomplished based on the left.fq and right.fq using Trinity [27] with min_kmer_cov set to 2 by default and all other parameters set to default.

Raw sequencing reads of TGS were filtered and subjected to Circular Consensus Sequences using the SMRT Analysis Server 2.20 (PacBio), and reads of inserts were obtained. After examining for poly(A) signal and 5′ and 3′ adaptors, and correction using proovread software [38], full-length and non-full-length cDNA reads were recognized. Finally, the redundancies were moved using CD-HIT-EST [22] to obtain unigenes.

### Gene functional annotation

All predicted protein-coding sequences were aligned using BLASTX to protein and nucleotide databases: using cutoff *E*-value ≤1e-5 with NCBI non-redundant protein (NR) NCBI non-redundant nucleotide (NT), Swiss-Prot protein (http://www.expasy.org/sprot/); COG (http://www.ncbi.nlm.nih.gov/COG/) using cutoff *E*-value ≤1e-3; protein family (Pfam: http://pfam.sanger.ac.uk/) using cutoff *E*-value ≤0.01; and KEGG (http://www.genome.jp/kegg) pathways using cutoff *E*-value ≤1e-10.

The Blast2GO program (http://www.blast2go.com) [65] was used to annotate GO (http://www.geneontology.org) terms based on the NR annotations (cutoff *E*-value ≤1e-6).

### Quantification of gene expression levels

Gene expression levels were identified by RSEM [27] for each sample. Clean data of Illumina were mapped onto the SMRT sequencing data, and read-count for each gene was obtained from the mapping results. Considering the effect of the sequence depth and gene length on the fragments, the read-count values for each gene were converted into FPKM value. Genes with FPKM > 0.3 in samples from two or more time points were selected for further analysis [29, 66, 67].

### Identification and function analysis of DEGs

Differential expression analysis was performed using the DESeq R package (1.10.1) [67] to identify DEGs between the salt-stressed and control samples, and between samples collected at different time points. DESeq provides statistical routines for determining differential expression in digital gene expression data using a model based on the negative binomial distribution. Resulting *P*-values were adjusted using the *p.adjust* function for controlling the false discovery rate. Genes with an adjusted *P*-value < 0.05 found by DESeq were assigned as differentially expressed, and the absolute value of log_2_(Group1/Group2) ≥ 1 was used as the threshold for determining significant DEGs between different time points.

The GO enrichment analysis of the DEGs was implemented using the GOseq R packages based on Wallenius non-central hyper-geometric distribution [68], which can adjust for gene length bias in DEGs. Finally, analyses of high-level functions and utilities of biological systems were carried out with the KOBAS [69] software to test the statistical enrichment of DEGs in KEGG pathways.

### Identification of putative TF and TP-related genes

All DEGs were aligned to a plant TF database (PlantTFDB4.0: http://planttfdb.cbi.pku.edu.cn) [35], and a relational database of cellular membrane transport systems (TransportDB: http://www.membranetransport.org; [36, 59]) to identify putative TFs and tTPs (*E*-value ≤1e-6), respectively.

### Validation of DEGs with qRT-PCR

The qRT-PCR assays were performed to validate the reliability of RNA-Seq analysis. RNA samples were used as templates for reverse transcription with the M-MLV RTase cDNA Synthesis Kit (TaKara, Dalian, China). Primers used in this study are listed in Additional file 15: Table S8. The expression of *actin2* gene was used as the internal control [14]. Real-time PCR was carried out with the SYBR Green PCR Master Mix system (Takara) on an Applied Biosystems 7500/7500 Fast Real-time PCR System (ABI, Foster City, CA, USA). The PCR amplification conditions were performed according to the methods described by Huang et al. (2017) [14]. Relative gene expression levels were calculated using the 2^–ΔΔ*C*t^ method [70].

### Statistical analysis

Results were based on three independent experiments with at least three replicates. The SPSS software package (ver. 17.0; SPSS Inc., Chicago, IL, USA) was used for statistical analysis. Significant differences among different time points for qRT-PCR and physiological index data were analyzed using one-way ANOVA with Duncan’s multiple range tests.

## Additional files

Additional file 1: Determination of physiological indices of *A. pumila* under 250 mM NaCl stress. (A) Chlorophyll content. (B) Proline content. (C) Malondialdehyde content. (D) Superoxide dismutase activity. (E) Na^+^ concentration. (F) K^+^ concentration. Data represents mean SE of three independent assays. Different lowercase letters represent statistically significant differences as determined by one-way ANOVA (*P* < 0.05, Duncan’s multiple range test). (TIF 1711 kb)

Additional file 2: Overview of sequence data quality obtained from Illumina sequencing. (DOCX 18 kb)

Additional file 3: Assessment of qualities of sequencing. (A) Comparison of transcript length distribution from PacBio RS and Illumina platforms. (B) Boxplot showing the length of unique genes in Illumina and SMRT. (TIF 1040 kb)

Additional file 4: Functional annotation and homology search of unigenes from SMRT sequencing dtata. (A) Venn diagram showing the number of common and unique genes annotated in five databases. (B) Species distribution of the result of NR annotation. (TIF 1283 kb)

Additional file 5: Summary of reads from the Illumina sequencing data and their matches in the SMRT sequencing data. (DOCX 17 kb)

Additional file 6: Comparison of gene expression levels under different experimental conditions. (A) Boxplot showing the distribution of FPKM values at seven time points of salinity stress. The X-axis in the boxplot is the sample name. The Y-axis is the log10(FPKM+ 1). (B) Number of genes expressed in each time point. (TIF 872 kb)

Additional file 7: Information on 8075 DEGs for different time points. (XLSX 7215 kb)

Additional file 8: Information on 4425 osmosis-related DEGs and 680 ion-related DEGs. (XLSX 4735 kb)

Additional file 9: Genes that were differentially co-expressed at all six time points. (XLSX 15 kb)

Additional file 10: GO terms for the 377 differentially co-expressed genes at time points 6, 12, 24 and 48 h. (TIF 1568 kb)

Additional file 11: Distribution of KEGG enriched pathways for the 377 DEGs at time points 6, 12, 24 and 48 h. The abscissa represents the richness factor reflecting the proportion of DEGs in a given pathway. The number of DEGs in the pathways is indicated by the circle area, and the circle color represents the range of the corrected *P* values. (TIF 1152 kb)

Additional file 12: The 483 differentially expressed transcription factors between different time points. (XLSX 461 kb)

Additional file 13: The 1157 differentially expressed transporter proteins between different time points. (XLSX 1136 kb)

Additional file 14: Twenty-one selected differentially expressed genes were validated by qRT-PCR assays. Comparison of RNA-Seq data (gray bar) with qRT-PCR data (red lines). The normalized expression (log10(FPKM+ 1)) of RNA-Seq is indicated on the Y-axis to the left. The relative qRT-PCR expression level is shown on the Y-axis to the right. (TIF 9983 kb)

Additional file 15: List of primers used in this study. (XLSX 13 kb)

## Abbreviations

*A. lyrata*: *Arabidopsis lyrata subsp. Lyrata*
*A. pumila*: *Arabidopsis pumila*
*A*. *thaliana*: *Arabidopsis thaliana*
ABC transporters: ATP-binding cassette transporters
ABCB1: ATP-binding cassette
*ALA1*: *Aminophospholipid ATPase 1*
CLC-A: A member of the voltage-dependent chloride channel
COG: Clusters of Orthologous Groups
CPK30: Calcium-dependent protein kinase
DEG: Differentially expressed genes
FPKM: Expected number of Fragments Per Kilobase of transcript sequence per Millions base pairs sequenced
GO: Gene Ontology
KEA2: K^+^/H^+^ antiporter 2
KEGG: Kyoto Encyclopedia of Genes and Genomes
KOG: EuKaryotic Ortholog Groups
KUP9: K^+^ ion transmembrane transporter 9
MAPK: Mitogen-activated protein kinase
*MAPKKK18*: *Mitogen-activated protein kinase kinase kinase 18*
MDA: Malondialdehyde
NAC: NAM/ATAF/CUG
NHX1: Na^+^/proton (H^+^) antiporter
NR: NCBI non-redundant protein
NT: NCBI nucleotide sequences
*P5CS1*: Delta1-pyrroline-5-carboxylate synthase 1
Pfam: Protein family
qRT-PCR: Quantitative real-time PCR
ROS: Reactive oxygen species
RSEM: RNA-Seq by Expectation Maximization
SGS: Second-generation sequencing
SMRT: Single-molecule real-time
SOD: Superoxide dismutase
SOS1: Salt Overly Sensitive 1
TF: Transcription factor
TGS: Third-generation sequencing
*Tnp2/En/Spm*: CACTA-like transposase family
TP: Transporter protein
VHA-A: A vacuolar ATP synthase
*VP1*: Vacuolar H^+^-pyrophosphatase
