# Supplementary figures and images for: Full-length transcriptome sequences of ephemeral plant Arabidopsis pumila provides insight into gene expression dynamics during continuous salt stress
Source: BMC Genomics. 2018 Sep 27;19:717. doi: 10.1186/s12864-018-5106-y (PMC6161380; doi:10.1186/s12864-018-5106-y)

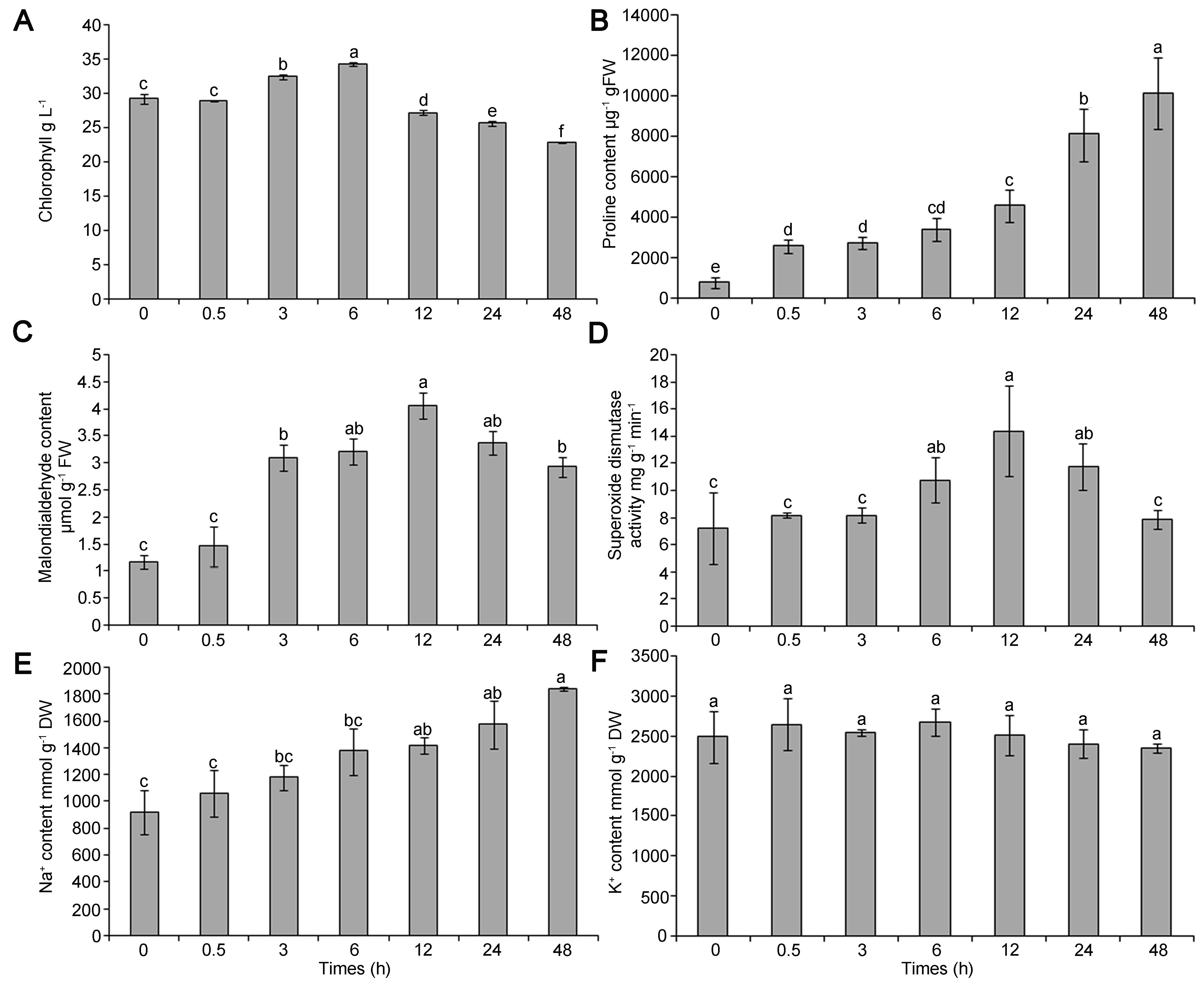

Supplement: Supplementary file 1 — Determination of physiological indices of A. pumila under 250 mM NaCl stress. (A) Chlorophyll content. (B) Proline content. (C) Malondialdehyde content. (D) Superoxide dismutase activity. (E) Na+ concentration. (F) K+ concentration. Data represents mean SE of three independent assays. Different lowercase letters represent statistically significant differences as determined by one-way ANOVA (P < 0.05, Duncan’s multiple range test). (TIF 1711 kb) [file 12864_2018_5106_MOESM1_ESM.tif]

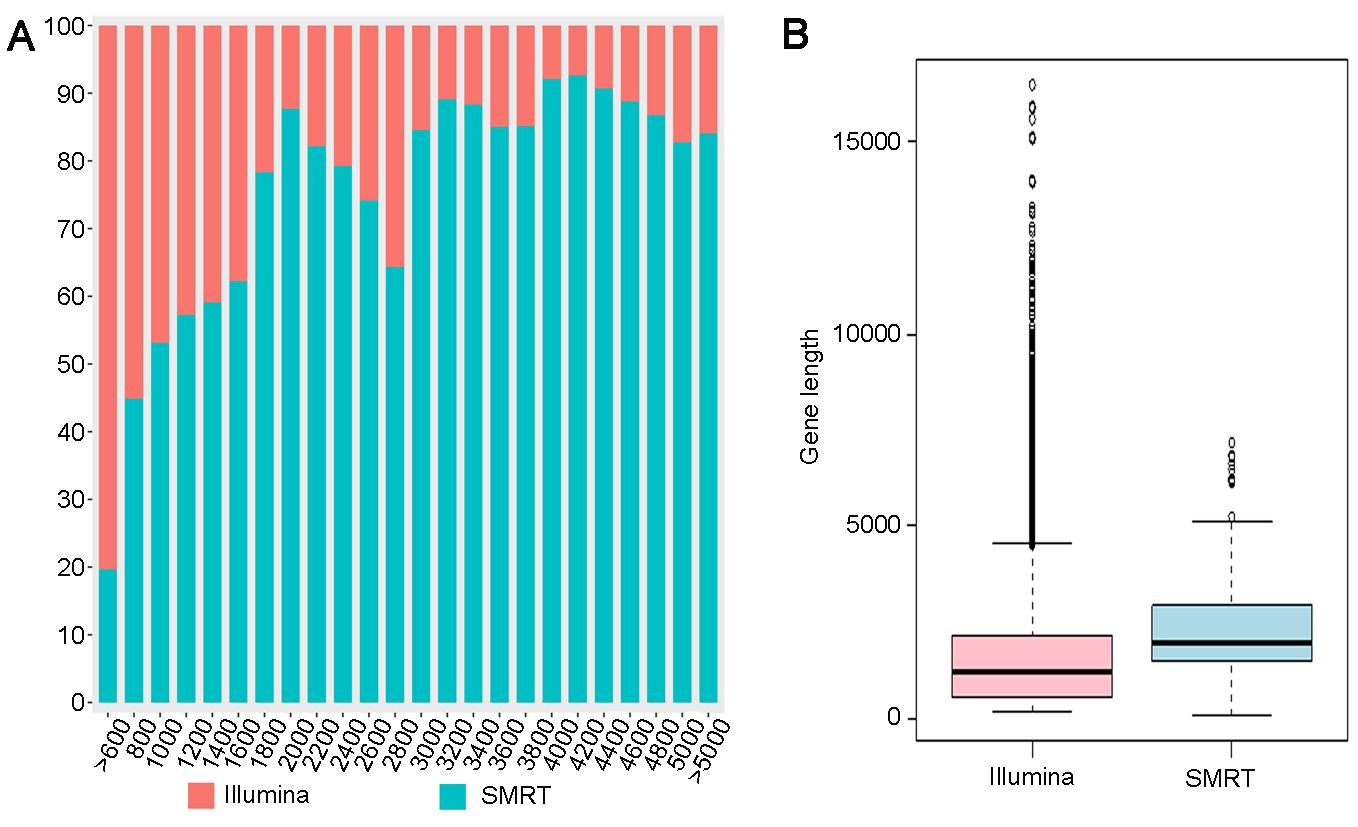

Supplement: Supplementary file 3 — Assessment of qualities of sequencing. (A) Comparison of transcript length distribution from PacBio RS and Illumina platforms. (B) Boxplot showing the length of unique genes in Illumina and SMRT. (TIF 1040 kb) [file 12864_2018_5106_MOESM3_ESM.tif]

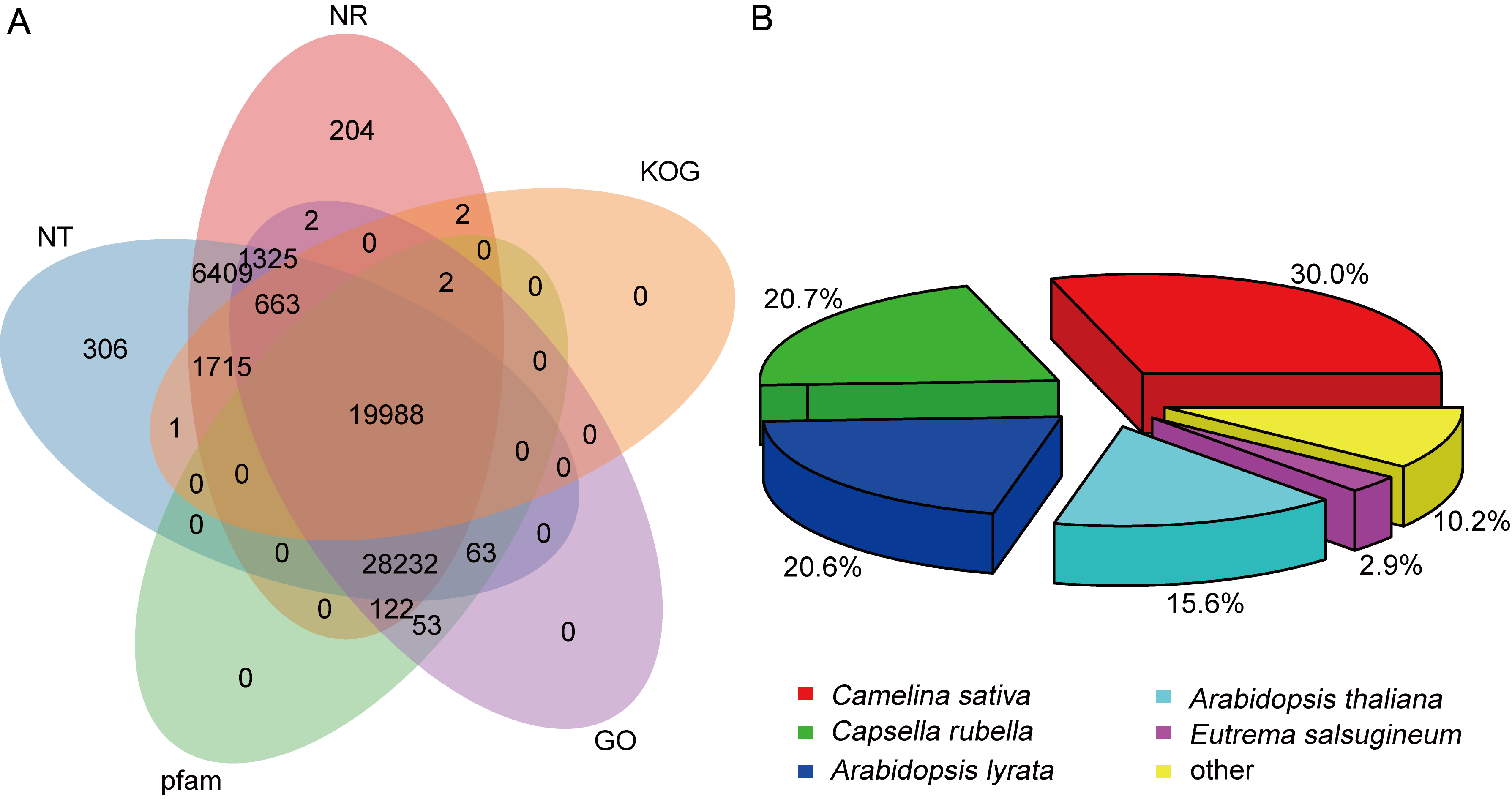

Supplement: Supplementary file 4 — Functional annotation and homology search of unigenes from SMRT sequencing dtata. (A) Venn diagram showing the number of common and unique genes annotated in five databases. (B) Species distribution of the result of NR annotation. (TIF 1283 kb) [file 12864_2018_5106_MOESM4_ESM.tif]

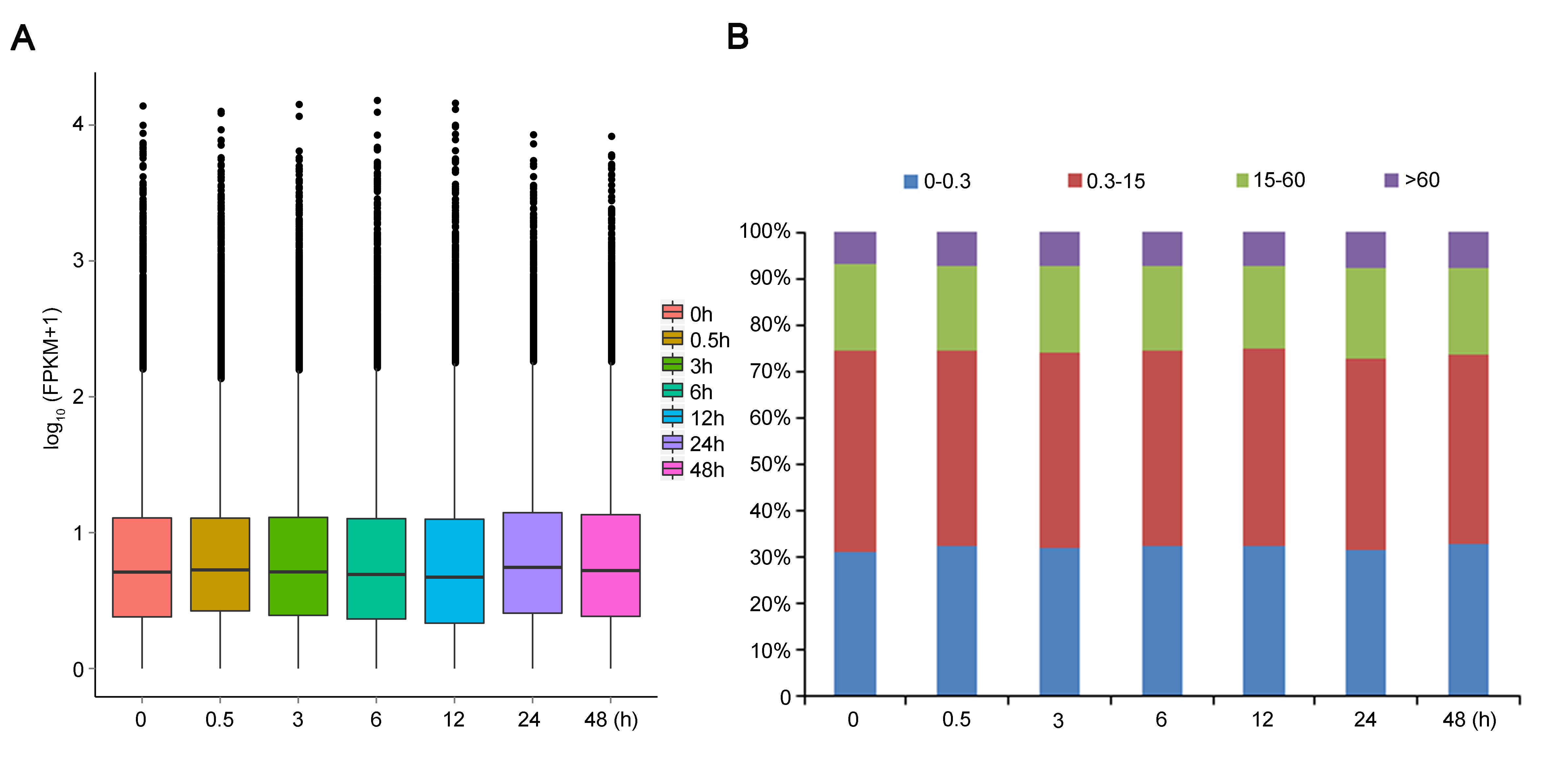

Supplement: Supplementary file 6 — Comparison of gene expression levels under different experimental conditions. (A) Boxplot showing the distribution of FPKM values at seven time points of salinity stress. The X-axis in the boxplot is the sample name. The Y-axis is the log10(FPKM+ 1). (B) Number of genes expressed in each time point. (TIF 872 kb) [file 12864_2018_5106_MOESM6_ESM.tif]

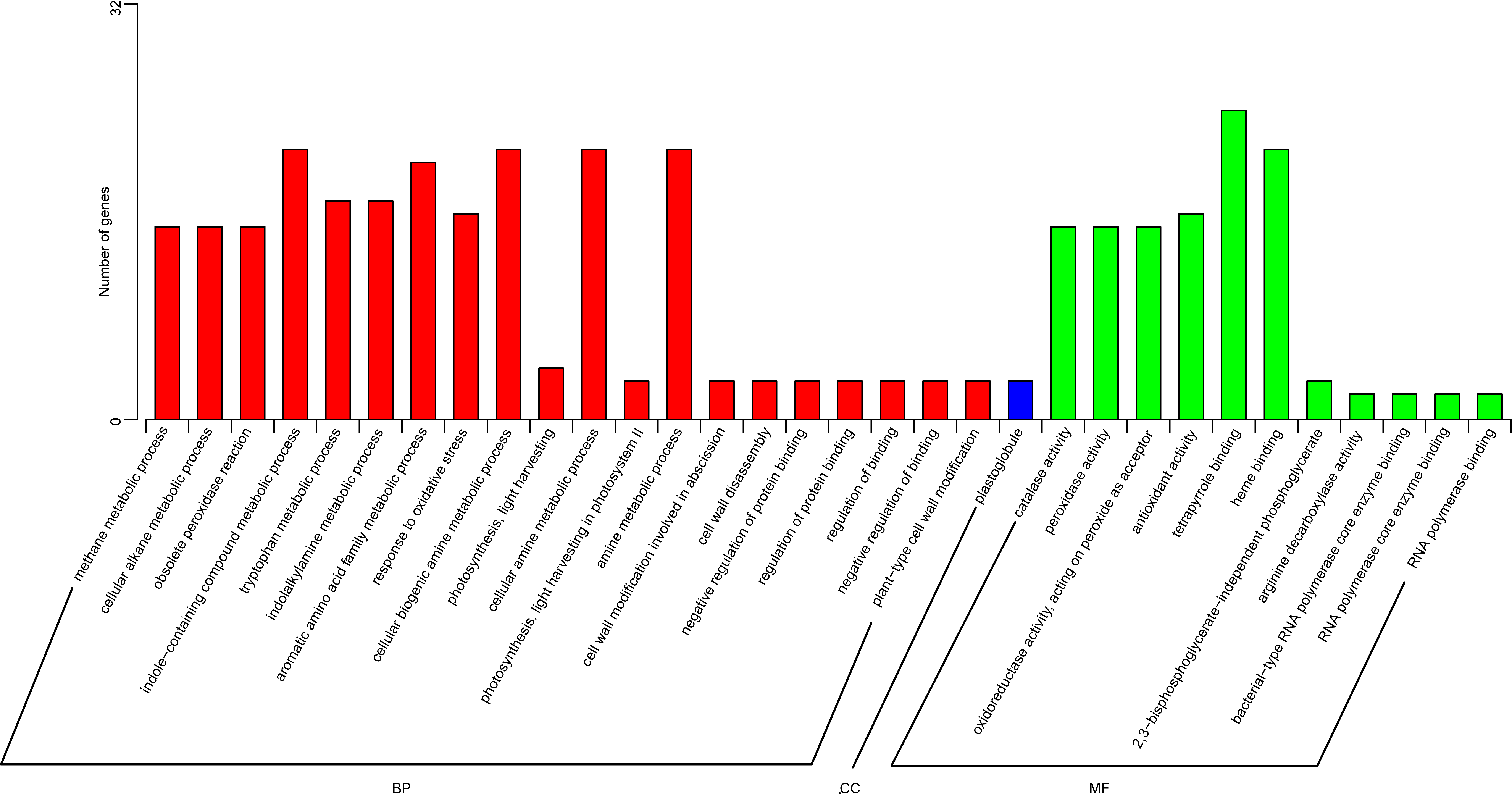

Supplement: Supplementary file 10 — GO terms for the 377 differentially co-expressed genes at time points 6, 12, 24 and 48 h. (TIF 1568 kb) [file 12864_2018_5106_MOESM10_ESM.tif]

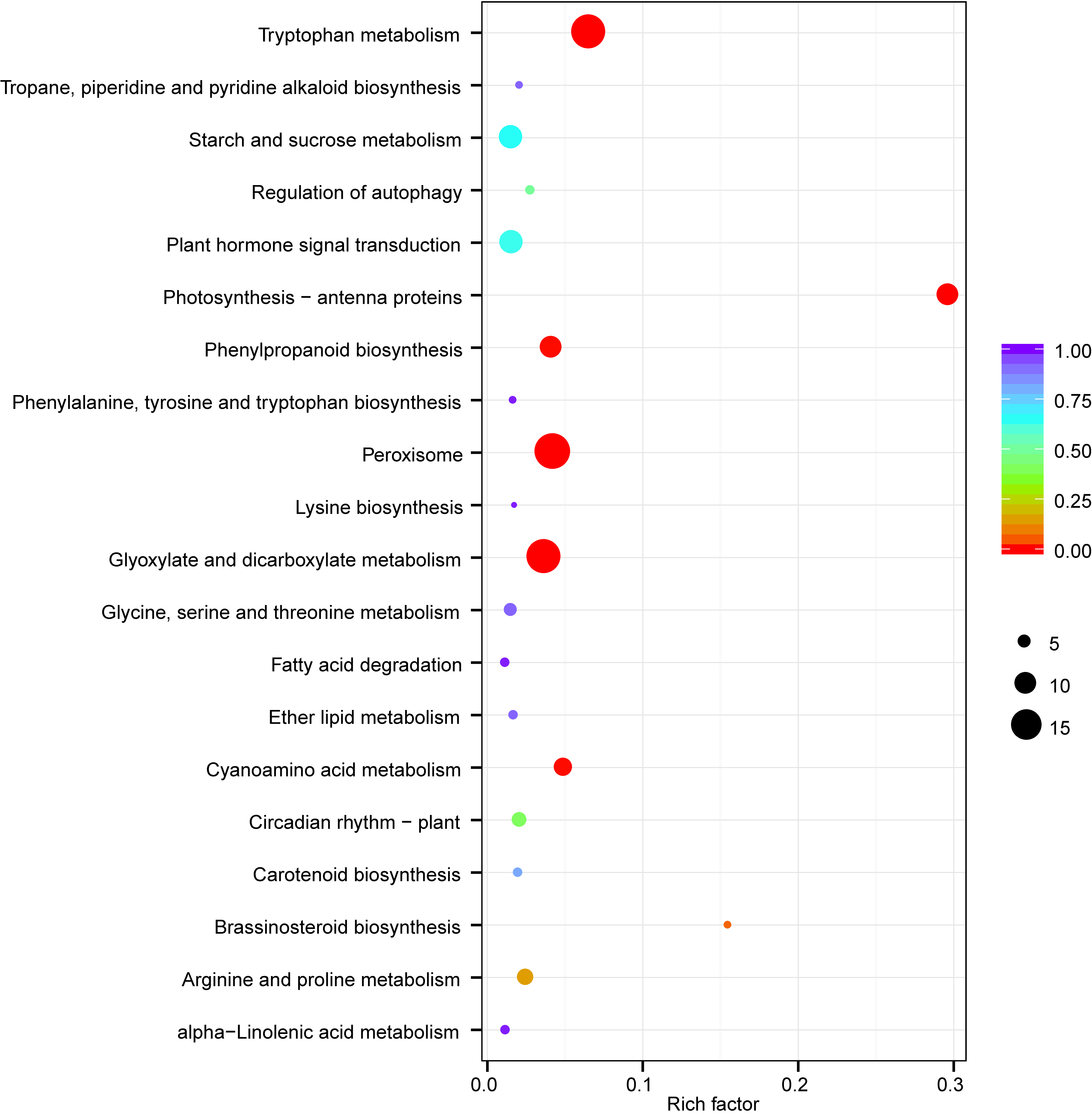

Supplement: Supplementary file 11 — Distribution of KEGG enriched pathways for the 377 DEGs at time points 6, 12, 24 and 48 h. The abscissa represents the richness factor reflecting the proportion of DEGs in a given pathway. The number of DEGs in the pathways is indicated by the circle area, and the circle color represents the range of the corrected P values. (TIF 1152 kb) [file 12864_2018_5106_MOESM11_ESM.tif]

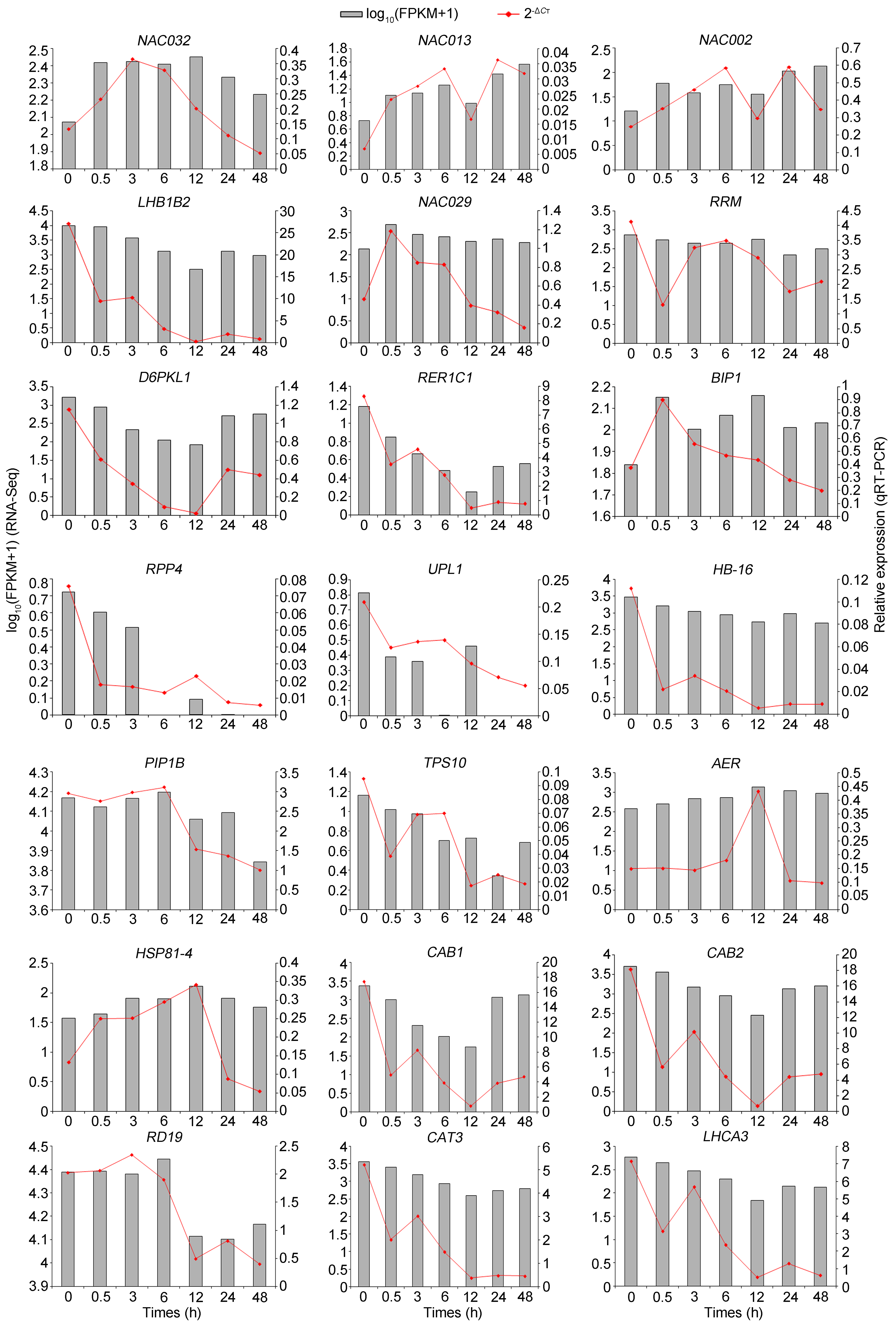

Supplement: Supplementary file 14 — Twenty-one selected differentially expressed genes were validated by qRT-PCR assays. Comparison of RNA-Seq data (gray bar) with qRT-PCR data (red lines). The normalized expression (log10(FPKM+ 1)) of RNA-Seq is indicated on the Y-axis to the left. The relative qRT-PCR expression level is shown on the Y-axis to the right. (TIF 9983 kb) [file 12864_2018_5106_MOESM14_ESM.tif]
