# Supplementary material for: Full-length transcriptome sequences of ephemeral plant Arabidopsis pumila provides insight into gene expression dynamics during continuous salt stress
Source: BMC Genomics. 2018 Sep 27;19:717. doi: 10.1186/s12864-018-5106-y (PMC6161380; doi:10.1186/s12864-018-5106-y)
Supplement: Supplementary file 2 — Overview of sequence data quality obtained from Illumina sequencing. (DOCX 18 kb) [file 12864_2018_5106_MOESM2_ESM.docx]

**Additional file 2: Table S1.** Overview of sequence data quality obtained from Illumina sequencing.

| **Samples** | **Raw Reads** | **Clean Reads** | **Clean Bases** | **Error (%)** | **Q20 (%)** | **Q30 (%)** | **GC Content (%)** |
| --- | --- | --- | --- | --- | --- | --- | --- |
| S0h_1 | 59096430 | 56842886 | 8.53G | 0.02 | 96.43 | 91.28 | 44.52 |
| S0h_2 | 61107124 | 58696786 | 8.8G | 0.02 | 96.35 | 91.09 | 44.75 |
| S0h_3 | 61660122 | 59408092 | 8.91G | 0.02 | 96.49 | 91.39 | 44.86 |
| S30min_1 | 46073742 | 44215738 | 6.63G | 0.02 | 97.23 | 92.74 | 43.93 |
| S30min_2 | 57510412 | 55333796 | 8.3G | 0.02 | 96.48 | 91.35 | 44.69 |
| S30min_3 | 46210570 | 44454100 | 6.67G | 0.02 | 96.21 | 90.8 | 44.65 |
| S3h_1 | 57705820 | 55135490 | 8.27G | 0.02 | 96.5 | 91.41 | 44.56 |
| S3h_2 | 55437056 | 53347370 | 8G | 0.02 | 96.53 | 91.47 | 44.61 |
| S3h_3 | 53931060 | 51864996 | 7.78G | 0.02 | 96.3 | 90.99 | 44.31 |
| S6h_1 | 62869010 | 60583026 | 9.09G | 0.02 | 96.52 | 91.42 | 44.47 |
| S6h_2 | 59271592 | 56766946 | 8.52G | 0.02 | 96.11 | 90.63 | 44.34 |
| S6h_3 | 60760922 | 58436848 | 8.77G | 0.02 | 96.51 | 91.44 | 44.36 |
| S12h_1 | 66101082 | 63681224 | 9.55G | 0.02 | 96.55 | 91.5 | 44.27 |
| S12h_2 | 60480902 | 58202434 | 8.73G | 0.02 | 96.58 | 91.57 | 44.29 |
| S12h_3 | 53696152 | 51567306 | 7.74G | 0.02 | 96.43 | 91.26 | 44.33 |
| S24h_1 | 58984264 | 56705498 | 8.51G | 0.02 | 96.52 | 91.44 | 44.33 |
| S24h_2 | 56508028 | 54321728 | 8.15G | 0.02 | 96.37 | 91.13 | 44.03 |
| S24h_3 | 66148852 | 63720516 | 9.56G | 0.02 | 96.37 | 91.14 | 44.31 |
| S48h_1 | 57226120 | 54879024 | 8.23G | 0.02 | 96.53 | 91.48 | 44.41 |
| S48h_2 | 42325506 | 40751522 | 6.11G | 0.02 | 96.41 | 91.23 | 44.26 |
| S48h_3 | 56808760 | 54627246 | 8.19G | 0.02 | 96.4 | 91.21 | 44.04 |
| **Total** | **1199913526** | **1153542572** |  |  |  |  |  |
