# Supplementary material for: Full-length transcriptome sequences of ephemeral plant Arabidopsis pumila provides insight into gene expression dynamics during continuous salt stress
Source: BMC Genomics. 2018 Sep 27;19:717. doi: 10.1186/s12864-018-5106-y (PMC6161380; doi:10.1186/s12864-018-5106-y)
Supplement: Supplementary file 5 — Summary of reads from the Illumina sequencing data and their matches in the SMRT sequencing data. (DOCX 17 kb) [file 12864_2018_5106_MOESM5_ESM.docx]

**Additional file 6: Table S2.** Summary of reads from the Illumina sequencing data and their matches in the SMRT sequencing data.

| **Samples** | **No. of cleaned reads** | **No. of mapped reads** | **Percentage (%)** | **No. of cleaned reads** |
| --- | --- | --- | --- | --- |
| S0h_1 | 56842886 | 38053086 | 66.69 | 56842886 |
| S0h_2 | 58696786 | 39478294 | 67.62 | 58696786 |
| S0h_3 | 59408092 | 40387068 | 67.98 | 59408092 |
| S30min_1 | 44063348 | 28127394 | 63.83 | 44063348 |
| S30min_2 | 55333796 | 37541678 | 67.85 | 55333796 |
| S30min_3 | 44454100 | 29746180 | 66.91 | 44454100 |
| S3h_1 | 55135490 | 36636710 | 66.45 | 55135490 |
| S3h_2 | 53347370 | 36981394 | 69.32 | 53347370 |
| S3h_3 | 51864996 | 35141396 | 67.76 | 51864996 |
| S6h_1 | 60583026 | 40322112 | 66.56 | 60583026 |
| S6h_2 | 56766946 | 37241672 | 65.6 | 56766946 |
| S6h_3 | 58436848 | 40504654 | 69.31 | 58436848 |
| S12h_1 | 63681224 | 43627826 | 68.51 | 63681224 |
| S12h_2 | 58202434 | 40067132 | 68.84 | 58202434 |
| S12h_3 | 51567306 | 35346178 | 68.54 | 51567306 |
| S24h_1 | 56705498 | 37729108 | 66.54 | 56705498 |
| S24h_2 | 54321728 | 34810386 | 64.08 | 54321728 |
| S24h_3 | 63720516 | 42744866 | 67.08 | 63720516 |
| S48h_1 | 54879024 | 35929862 | 65.47 | 54879024 |
| S48h_2 | 40751522 | 27006406 | 66.27 | 40751522 |
| S48h_3 | 54627246 | 36021322 | 65.94 | 54627246 |
| **Total** | **1153390182** | **773444724** |  |  |
